# General practitioners' and district nurses' conceptions of the encounter with obese patients in primary health care

**DOI:** 10.1186/1471-2296-12-7

**Published:** 2011-02-19

**Authors:** Lena M Hansson, Finn Rasmussen, Gerd I Ahlstrom

**Affiliations:** 1 Department of Public Health Sciences, Karolinska Institute, Norrbacka floor 5, 171 76 Stockholm, Sweden; 2 The Swedish Institute for Health Sciences, Lund University, Box 187, 221 00 Lund, Sweden

## Abstract

**Background:**

Primary health care specialists have a key role in the management of obesity. Through understanding how they conceive the encounter with patients with obesity, treatment may be improved. The aim of this study was thus to explore general practitioners' and district nurses' conceptions of encountering patients with obesity in primary health care.

**Method:**

Data were collected through semi-structured interviews, and analysed using a phenomenographic approach. The participants were 10 general practitioners (6 women, 4 men) and 10 district nurses (7 women, 3 men) from 19 primary health care centres within a well-defined area of Sweden.

**Results:**

Five descriptive categories were identified: Adequate primary health care, Promoting lifestyle change, Need for competency, Adherence to new habits and Understanding patient attitudes. All participants, independent of gender and profession, were represented in the descriptive categories. Some profession and gender differences were, however, found in the underlying conceptions. The general staff view was that obesity had to be prioritised. However, there was also the contradictory view that obesity is not a disease and therefore not the responsibility of primary health care. Despite this, staff conceived it as important that patients were met with respect and that individual solutions were provided which could be adhered to step-by-step by the patient. Patient attitudes, such as motivation to change, evasive behaviour, too much trust in care and lack of self-confidence, were, however, conceived as major barriers to a fruitful encounter.

**Conclusions:**

Findings from this study indicate that there is a need for development and organisation of weight management in primary health care. Raising awareness of staff's negative views of patient attitudes is important since it is likely that it affects the patient-staff relationship and staff's treatment efforts. More research is also needed on gender and profession differences in this area.

## Background

The number of obese adults in Sweden has doubled over a 20-year period, and about 10% of both women and men are estimated to be obese [1]. Recent data show that the prevalence of obesity in a primary care setting is 20-24% [2]. In Sweden, teams of general practitioners (GPs) and nurses, especially district nurses (DNs), are on the front line of the encounter between health care personnel and people with obesity or other lifestyle-related diseases [3]. Consequently, these professional groups must, to a greater extent than before, assist patients with weight management. The trend in health care is towards a more patient-centred approach, empowering the patients themselves to take responsibility for their health [4]. Accordingly, one of the tasks of GPs and DNs is to support patients in making healthy decisions about their daily living habits.

Primary health care is recognised as an important resource in co-ordinating the treatment of obese patients [5]. Previous studies also reveal that GPs and nurses acknowledge that primary care has an important role in managing obesity [6,7]. Other studies, however, show that GPs do not regard obesity management as their responsibility, but rather that of the patient [8].

A significant proportion of GPs and nurses in primary health care consider themselves insufficiently skilled or prepared to treat patients with obesity [6,7]. Both GPs and nurses regard themselves as ineffective in their work with obese patients [7,9], and a majority of GPs consider the possibility of a patient's succeeding with weight loss as limited [6,9]. Studies have also shown that medical staff attribute the failure to lose weight to personal, patient-related factors rather than to professional ones [8,10]. Several other studies support this notion, since they show that GPs and nurses perceive obese patients as having low motivation, lacking willpower, being unwilling to change lifestyle [11,12] and non-compliant to advice [10]. However, there are studies supporting the presence of system-level barriers, such as lack of time during patient visits [12], lack of places to which to refer patients, and lack of patient-oriented educational materials [13].

The seemingly negative beliefs about obesity and obesity treatment which have been documented, especially in quantitative studies, might be over-simplified, and it is likely that GPs and nurses hold more complex views. Qualitative studies of GPs and nurses in primary health care support this idea to some extent [8,12,14]. Nurses perceive weight as a sensitive issue to discuss with patients and therefore try to achieve a balance between factors involving personal responsibility and factors outside the individual's control [14]. Mercer and Tessier found that GPs were negative about their role in obesity treatment, while practice nurses, although accepting it as part of their job, experienced that GPs "off-loaded" patients on to them [12]. Epstein and Ogden found that GPs were sceptical about the success of available treatments, but still offered anti-obesity drugs and tried to listen to and understand the patient's problem [8].

Reviewing the literature on existing qualitative studies, including those of nurses and GPs in primary health care, it becomes apparent that there is a need to know more about how they conceive their encounters with patients with obesity. Research has hitherto been limited in that no male nurses have been included, despite its having been demonstrated that gender might be important regarding attitudes and practices within obesity management [9,15]. This emphasises the need for further research. Against this background, the aim of the current study was to describe how GPs and DNs, both male and female, conceive their encounters with obesity in primary health care.

## Methods

We used a phenomenographic approach to identify and describe the various ways in which a specific phenomenon is conceived [16], in this case primary health care specialists' conceptions of obesity and obesity treatment. This qualitative approach has been used in health care research to study, for example, doctors' or nurses' conceptions of treatments for different diseases [17]. The phenomenographic tradition acknowledges two research perspectives: there is a first-order perspective which concerns how the world actually is, and a second-order perspective which concerns how the world is experienced. In phenomenography, the second-order perspective is essential [16]. The assumption is that there is only one world, but that it can be experienced in qualitatively different ways. The approach comes from educational research and entails adopting a content-related perspective to characterise, understand and describe the qualitatively different ways in which people make sense of the world around them. The conceptions are derived from individual interviews, but the analyses emanate in descriptive categories at a collective level [16].

### Participants

Eligible for participation in the study were staff from 57 primary health care centres in a well-defined area in Sweden. The criteria for inclusion were being a DN or GP with a specialist education, speaking Swedish fluently, and acceptance of tape-recording. A strategic selection of participants was made to obtain variety regarding age, gender and primary health care experience. This would provide a range of conceptions from DNs and GPs. The heads of the primary health care centre in question had to give their consent for staff to participate. Therefore we first contacted the heads by e-mail with an information letter, then by telephone approximately a week later. If the head gave permission, the informant in question was approached by either e-mail or telephone. We gave the informants the same information as their head.

We included 20 participants at 19 primary health care centres (Table 1). Two DNs came from the same centre. The participants' median age was 51 years, and their median professional experience 10.5 years. Of the 20 informants, 10 were recommended by their medical head, 3 were medical heads themselves, working as GPs, and 7 were contacted from staff lists. Four of the 10 DNs were specialists in diabetes or weight management. Among the GPs, 3 were involved in some kind of weight management strategies.

**Table 1:** Demographic characteristics of the primary health care professionals (n = 20).

Characteristics	General practitioners	District nurses
	*n *= 10	*n *= 10
Sex		
Female	6	7
Male	4	3
Age		
34-40	2	1
41-45	2	2
46-50	2	2
51-55	1	2
56-60	3	3
Years in profession		
1-5	4	2
6-10	2	2
11-15	2	2
16-25	-	2
26 or more	2	2
Country of birth		
Sweden	9	9
Other	1	1

The medical heads at 16 primary health care centres refused to grant permission for participation, largely because of re-organisation, work overload or shortage of staff, although some were simply unwilling. We could not reach the medical heads at 10 centres. At 12 centres the medical head gave approval for the study but the DNs and GPs declined because of work overload, unwillingness or lack of financial compensation; some could not be reached. The Regional Ethical Review Board, Karolinska Institute, Stockholm, Sweden, approved the study (reference number 2006/7-31/1).

### Data collection

Two of us (LMH, GA) developed an interview guide with open-ended questions. The main questions were: *What are your experiences of meeting patients with obesity? How do you conceive the life situation of patients with obesity? How do you think care is working for patients with obesity? *Individual interviews, which took the form of conversations, were performed by one of us (LMH). Follow-up questions were asked depending on how comprehensively the informant answered the main questions. Examples of follow-up questions were: "What do you mean?" "Can you explain?" "Can you tell me more?" "Is there anything you would like to add?" Participants chose where the interview should take place. Two of the 20 participants preferred to be interviewed at the interviewer's research department, while the remaining interviews were performed at the workplace. The tape-recorded interviews (30-80 minutes) were transcribed verbatim.

### Data analysis

We carried out the analysis in four steps in accordance with the phenomenographic approach [18,19]. At the first step, the interviews were listened to again to verify that they had been transcribed correctly. The individual transcripts were then read through several times to get an overall impression, and thereafter statements relevant to the study were identified. At the second step, the aim was to identify distinct ways of conceiving obesity treatment, which involved constantly comparing and labelling the statements. The labelled statements were then treated as preliminary conceptions. At the third step the conceptions were compared with one another and grouped to obtain an overall picture of possible links between them. The grouped conceptions formed preliminary descriptive categories to which names were given. At the fourth step, the focus shifted from relations between conceptions to relations between the preliminary descriptive categories. Finally, the descriptive categories were critically investigated to ensure that they properly represented the conceptions.

## Findings

There emerged five descriptive categories illustrating GPs' and DNs' conceptions of their encounters with obese patients in primary health care (Table 2). The conceptions in each descriptive category are illustrated by quotations from individual interviewees.

**Table 2:** Staff's conceptions of encounters with patients with obesity in primary health care.

Perspective	Descriptive categories	Conceptions	Number of statements	Number of interviews
Organisational	Adequate primary health care	Overweight needs to be prioritised	80	1-7,9-20
		Lack of distinct guidelines and evidence	70	3-5,7-11,14-20
		Overweight not our responsibility	50	1,3-9,11-17,19
		Continuity and long-term support	31	1,3-5,8,10,11,13,14,16-20
		Co-operate for knowledge-based care	27	1,2,4-7,10,12,14-16,18
Staff	Promoting lifestyle change	Small steps and realistic goals	99	1,3-20
		Raise awareness	67	1,3-8,10-20
		Individually based solutions	55	1,3-6,8-11,13-15,17-20
		Facilitate motivation	53	2-16,18-20
	Need for competency	Respectful encounters	53	2-6,8,10-15,17-20
		Staff with active interest	43	2-17,19,20
		Knowledge about diet and counselling	39	1-5,7-10,12-19
Patient	Adherence to new habits	Overcome deep-seated habits	69	1-3,5-13,15-20
		Psychological and medical barriers	43	1,2,5,7-15,17-19
		Socio-cultural barriers	27	1,2,6-8,11-15,18-20
	Understanding patient attitudes	Motivation to change	131	1-20
		Evasive behaviour	73	1-4,6-18,20
		Trusting in care	64	1,3-7,9-13,15-20
		Lack of self-confidence	17	2,6,8,10,14,15,19

All participants, independent of profession and gender, were represented with statements in each descriptive category. However, certain profession and gender differences were identified regarding the conceptions forming the descriptive categories. If more than two thirds of all the statements within a conception were made by one of the compared groups (gender or profession), this is reflected in the account of the findings.

### Adequate primary health care

#### Overweight needs to be prioritised

Staff regarded primary care as having an important role to play in both the prevention and treatment of overweight and obesity. However, there was a major barrier to working effectively in this area, namely lack of time. Sometimes staff avoided bringing up weight issues because they considered that they would not have time to deal properly with the problem later on. DNs often devoted more time per visit than GPs, but experienced that there were competing assignments hindering them from working more actively with these patients. Staff considered that GPs had much less time because more easily treated conditions were often prioritised. They regarded the system of reimbursing health care centres as in need of improvement.

*"We have no resources and that's why we can't do anything. There's no time. We should actually devote more time to prevention, but it's all about diseases." *(DN, female, 58 years old)

#### Lack of distinct guidelines and evidence

The lack of guidelines and evidence within the area of obesity was more strongly experienced by the men than by the women. The view of male DNs, for instance, was that there are few treatment options to offer the patients. Also, the existing methods, except for surgical intervention, were considered to be ineffective and not evidence-based. Staff were especially eager for guidelines regarding dietary advice, which at present tended to be vague. Because of the many contradictions, different opinions and extensive debate about what were the most successful diet regimes, staff regarded it as difficult to offer balanced advice to patients. The diet pills and medical treatments available for losing weight were also considered ineffective. Staff might be prepared to recommend them but were not optimistic about the outcome.

*"I feel I don't have much to offer medically. It's very general advice at best, and a recommendation to try Weight Watchers or something like that. For those who've tried everything, it might be a referral for surgical treatment." *(GP, male, 38 years old)

#### Overweight not our responsibility

This conception has to do with staff's reporting that the treatment of overweight and obesity should not self-evidently be performed within primary health care. This conception was more pronounced among GPs than DNs. However, female DNs were more inclined than male DNs to deny that primary health care plays an important role. Staff considered that their main task was to treat diseases, and overweight and obesity were seen more as conditions that might involve a risk of diabetes or some other disease. If a concomitant disorder was present, however, it was important to intervene. GPs, though, thought that other groups within primary health care, such as DNs, physiotherapists and dietitians, could handle this better than members of their own profession. Commercial weight-loss organisations were also thought to be more effective, and therefore more appropriate. Furthermore, staff thought that overweight and obesity constituted a societal problem, where community planning, school policies and information to parents were what mattered most. Some staff, however, acknowledged that health care had a role to play in prevention, but this only applied to child health care centres and school health services.

*"I don't think you should take it for granted that we're the ones to intervene. We're trained in medical care. Overweight and obesity are more of a societal problem." *(GP, male, 49 years old)

#### Continuity and long-term support

This conception has to do with staff's belief that obesity must be managed over an extended period. Staff emphasised continuity in care because lifestyle change was conceived of as taking time. Often, they saw that people were non-compliant to advice year after year but then suddenly things started to happen. The importance of encountering the same personnel was stressed by a number of staff. For the patients, this would mean not having to repeat their story again and again, and would also mean not being given contradictory information.

*"It has to be a long-term relationship. Often it's very short encounters, but I've noticed that I can get further with the patients I meet repeatedly." *(DN, male, 55 years old)

#### Co-operate for knowledge-based care

Staff considered it important to adopt a team approach, which included different competencies. Co-operation had to be both within and outside primary health care. Staff regarded psychologists, welfare officers and psychiatrists as especially important collaboration partners, as some patients had eating problems or other psychological problems. Patients were often referred to centres specialising in obesity management, but staff saw little real co-operation taking place. Otherwise, co-ordinated efforts in the local community, where primary care would be just one of the stakeholders, were seen as an important next step in improving care.

*"Well, what we have, and I think is very positive, is access to a dietitian, helpful doctors, a social welfare officer and a nurse competent in cognitive therapy. I think we can solve this in-house." *(DN, female, 55 years old)

### Promoting lifestyle change

#### Small steps and realistic goals

Staff regarded it as necessary to help patients find ways to improve lifestyle. The focus was mainly on increasing physical activity, especially lighter activities on a day-to-day basis, like cycling or walking to work. To recommend more intense workouts, like running, was seen as counter-productive, because patients were unlikely to adhere to such a regime. Some staff put exercise on a prescription basis (whereby, for example, the patient had the right to go to a gym during working hours), because they thought it gave additional force to their advice. However, some experienced that this worked best with patients who were already motivated but had not yet started, or who had done some exercise before. Staff also considered it important to encourage patients to reduce their energy intake. However, staff saw it as important to advise the patient to take small steps and start with one thing at a time. Often, patients were eager to go ahead and make changes in all areas at once. Unrealistic expectations about weight loss and progress had prompted staff to help patients find more realistic and achievable goals.

*"I try to get them to be active on a daily basis. Walking a short distance to work or using the stairs. It's important that they begin changing their behaviour slowly." *(DN, female, 56 years old)

#### Raise awareness

Staff considered that patients were not always aware of their weight status or unhealthy lifestyle. Obese patients often sought care for neck, back and knee pain, heart and lung problems, or for general tiredness. Therefore it was important to give factual information about the association between obesity and ill-health. It was also regarded as helpful to use self-report questionnaires on health and lifestyle, or food diaries, as a basis for talking about the patient's condition. Staff considered that many did not reflect at all on their dietary patterns, and that the recording of food intake made patients more attentive. Staff also relied on medical health data, which constituted very straightforward information. One strategy to raise weight awareness was to measure body size or weight and then to move on to presenting facts about the association with ill-health.

*"I think these general health questionnaires about smoking, physical activity, diet and alcohol are a way of approaching the weight issue. They might not even have thought about the fact they don't eat vegetables every day." *(GP, female, 45 years old)

#### Individually based solutions

Staff regarded it as important to start from the patient's perspective and keep as open a mind as possible. They also considered it important to ask a lot of questions and to take careful note of the thoughts and strategies that the patient had applied in the past or was now applying. It was important to help patients find their own solutions, the staff's task being to guide the patient towards the right level of ambition. Different solutions were required for different patients. Contrary to what staff sometimes expected, patients with fewer resources might make the best progress. Staff experienced that solutions could always be found, and that these solutions could be managed by the patients. However, in more difficult cases, staff on occasion told patients that it might be time to stop trying to lose weight and focus on something else.

*"It's obvious that it has to be adapted to the individual. It's very personal what work, how old they are, etc. It may not even be a question of how much food they eat, but what and when they eat." *(GP, female, 34 years old)

#### Facilitate motivation

It was important for staff to emphasise the improved health that could be achieved by changing eating habits and physical activity regardless of any weight loss. Showing patients' improvements on health indicators was also regarded as motivational. Female GPs and DNs highlighted this to a greater extent than male GPs and DNs. Furthermore, staff considered that group activities were very useful. Not only the exchange of experience, support, and company, but also the pressure that could be exerted within the group enabled people to take responsibility. However, some staff considered that there were cases where more drastic methods were needed, like pushing the patient quite hard, making strong demands or using scare tactics. Staff told patients that they were going to have severe complications, develop certain diseases, or could even die from obesity.

*"I sometimes say: 'Heart disease, do you want that? Or diabetes?' I try to scare them a little bit and if I find out that their mother died of a heart attack I can use that." *(DN, female, 50 years old)

### Need for competency

#### Respectful encounters

Staff were aware of the stigmatisation of obese people, and also of the fact that it was hurtful. They therefore tried to be sensitive when raising issues of lifestyle and weight, and were careful not to blame the patients themselves. When medical advice had been sought concerning conditions not related to weight, but staff still raised the weight issue, some patients were offended and angry, even furious. For this reason, staff often waited until the second or third encounter before raising the matter. They sensed they had to establish a good relation in order not to seem provocative and possibly lose trust. Moreover, staff tried to show empathy and an understanding of the difficult situation of being obese. Often, staff perceived that the patient needed to be consoled, emotionally supported and acknowledged. Feelings of shame and guilt were often apparent. The hopelessness that some patients described was also difficult to handle, but staff tried to provide encouragement and hope. Female GPs made greater reference to respectful encounters than the other groups.

*"It's very much a question of comforting words or, so to speak, off-loading the blame. You need the right psychological feeling for meeting these individuals and their giant dilemma." *(GP, female, 44 years old)

#### Staff with active interest

This conception has to do with staff's interest in weight management. Staff considered that work with overweight and obese patients was best performed by those with an active interest in the area. An enthusiastic person with a strong driving force who really had a commitment was perceived as enhancing the patient encounter. GPs considered that nurses were often very interested in helping obese patients and actively trying to improve their work in this respect. DNs regarded the work as part of their profession and had an independent interest. DNs' conceptions of GPs' involvement were divided. Some DNs regarded GPs as initiators of weight management, others regarded them as not particularly interested, and sometimes evasive. Some GPs expressed a willingness to work with overweight and obesity but accepted that their colleagues were often not eager to get involved.

*"The nurses are probably a bit more oriented towards this kind of work, and I think they can do a lot. They have more time to go through things, and they're highly competent. Anyway, the ones we have have taken an active interest." *(GP, male, 52 years old)

#### Knowledge about diet and counselling

This conception has to do with how staff regarded training in different counselling techniques and basic knowledge about diet as essential to their encounters with obese patients. Staff considered that they needed pedagogic skills to help patients take decisions about lifestyle. They also experienced that special education in motivational interviewing and cognitive behavioural techniques would make for more fruitful encounters. However, many of the staff wanted better knowledge about the basic principles of nutrition and what constitutes a healthy diet. Staff tried to follow the constantly ongoing debate and catch up with new findings about successful dietary interventions, but expressed a need for short training courses.

*"Our basic and further education has to become much better. First, there's all this discussion about what diet to recommend, and second, about how to get people to do what you tell them*. (DN, male, 58 years old)

### Adherence to new habits

#### Overcome deep-seated habits

Staff regarded it as very difficult for people to change their lifestyle. They indicated that very few succeed in losing weight, and even those only lose a little. Lack of success among their patients has also made them less optimistic about their ability to help others in the future. Patients were perceived as having tried a huge set of strategies to lose weight, but without result. Staff attributed this largely to patients' difficulty in changing their behaviour. Lack of knowledge was not an issue for most patients, but the question was how they were going to put their knowledge into practice. Often, patients managed their new daily habits for some months but then relapsed. The view was that it is too tough to stick to a new regime and that the lack of an immediate positive outcome made people give in.

*"It's not so easy to change old routines. I think most people know how to eat, but it's one thing to know how and another thing to actually do it." *(DN, female, 55 years old)

#### Psychological and medical barriers

This conception concerns how staff regarded patients as facing psychological, medical and physical barriers to adhering to new habits. Males reflected more on this than females. The view was that pain in knees and hips limited opportunities for physical activity. Diabetes was often part of the picture, and this was regarded as requiring the patient to be extra compliant to advice about losing weight. Some staff also indicated that their patients had to rely on many drugs, which made it difficult to adopt new lifestyle habits. Otherwise, patients who were perceived as having psychological or psychiatric problems (depression, anxiety or addiction) were regarded as especially non-compliant. Often, they used food as a way of handling their distress, and changing eating behaviour was especially difficult. Medications against depression and anxiety were also regarded as causing weight gain, which discouraged patients from improving their habits.

*"Often orthopaedic problems hinder people and one thing leads to the other. Orthopaedic problems increase and then you can't move around. Your weight goes up and of course it gets harder to exercise." *(GP, male, 52 years old)

#### Socio-cultural barriers

This conception has to do with staff's considering that their patients were facing social and cultural barriers in adhering to new habits. Working hours, family life and financial situation were perceived as important factors affecting adherence, whilst patients from other cultures had the additional problem of finding appropriate food options corresponding to the traditions in their home countries. Staff sometimes advised moderate exercise, like swimming, to very heavy patients, but this could involve the problem of having to undress in front of strangers. DNs, especially the male DNs, indicated that socio-cultural barriers may have an important influence on behaviour.

*"There are quite a lot here that come from Asia and the Mediterranean area and they often have dinner very late and have particular eating habits. It's very difficult to make them change things." *(DN, male, 35 years old)

### Understanding patient attitudes

#### Motivation to change

Staff experienced that it was the patients' own responsibility to find the motivation. Patients had to come up with their own ideas about what to do. Willpower was very important. Patients had to commit themselves, be prepared to put in a lot of work, and make weight their first priority. However, some patients were described as a bit lazy, lacking in energy and indifferent to their situation. Some were regarded as having the motivation to lose weight but still reluctant to make the necessary changes. Those who sought professional care to lose weight were sometimes regarded as just wanting to wear smaller clothes. Even though some slight physical symptoms were often present, their main motivation was a better appearance. Staff considered that patients often experienced ashamed of their appearance, and that the low degree of acceptance of larger bodies in society acted as a motivator for losing weight. However, some staff thought the opposite, namely that there is little motivation to lose weight because so many others in society are just as obese. Patients who had experienced, or had close experience of diabetes, a heart attack or other severe problem were often highly motivated to do something. Mild back pain or a hurting knee could also be motivating factors, but some staff considered that many patients just adapted to their excess weight and did not seek help before they experienced very uncomfortable.

*"Patients want to lose weight but they don't want to change. Start walking instead of taking the bus, and eat less, that's all there is to it. Or the motivation might be there but they don't really want to do it, only if they think it's important." *(GP, male, 49 years old)

#### Evasive behaviour

Staff indicated that patients with obesity often made excuses for not coming to appointments, following advice or taking exercise. Patients tended to blame their failures on such things as family problems, lack of time, lack of money, and sometimes pain and medication. These patients were also regarded as having a tendency to disappear without notice. They were compliant for some time but then made themselves unavailable for follow-up. DNs were often the ones taking care of the follow-up and this group expressed greater concern about patients' evasive behaviour than GPs. Furthermore, what the staff perceived as very striking about this group of patients was that they claimed not to eat much and exercise a lot, and yet did not lose weight. Here it was very difficult for staff to find ways of telling the patient that this was in conflict with scientific evidence.

*"They often say 'I don't understand it, I don't eat anything', but actually we know they do." *(DN, female, 45 years old)

#### Trusting in care

Staff considered that many patients sought medical care just to get diet pills. They had tried different methods, and now their hope lay in pills or other medical treatment. The patients wondered if they suffered from some metabolic disturbance and wanted GPs to do tests. However, staff stated that the tests were seldom positive. Some patients also turned to staff in the hope that they would come up with a solution that worked. Staff regarded patients as off-loading their problem, and as expecting them to see to it that the excess weight disappeared as if by magic. Staff regarded patients as unwilling to assume the responsibility for losing weight, transferring it instead to the GP or DN.

*"I think a lot of them believe that someone else is going to do the job for them.... They put the responsibility on me, I'm the one who's going to fix it so they lose weight. I try to talk them out of it, but some don't listen." *(DN, female, 59 years old)

#### Lack of self-confidence

Female staff (and one male GP) experienced that patients lacked self-confidence in their ability to lose weight and adopt a healthy pattern of behaviour. Patients were regarded as being highly motivated but also expressed hopelessness about the fact that, even though they had tried a huge number of strategies, they had not succeeded in losing weight. Staff considered that patients' disappointment at not managing their overweight made them lose confidence, and feelings of guilt, frustration, despair and low self-esteem were often apparent.

*"There comes a time when you get so disappointed with yourself, because you just can't lose weight. You think you've done everything, and you still can't like yourself. You lose confidence." *(GP, female, 35 years old)

## Discussion

We found a wide variation in GPs' and DNs' conceptions of their encounters with obese patients in primary health care. Staff described the encounters from both an organisational/personnel perspective and a patient perspective. The need for primary care to have an adequate organisation for obesity treatment and competent staff for promoting lifestyle change was stressed. However, patients' adherence and attitudes to behaviour change were also looked upon as important. In addition to the findings on the collective level we found certain differences in the pattern of conceptions according to profession and gender. However, in view of the small number of male participants, especially in the DN group, these findings have to be interpreted cautiously.

The findings of the present study are to some extent in line with those of previous quantitative and qualitative investigations of attitudes and beliefs regarding obesity treatment in primary care. Examples of such beliefs are that primary care is not an entirely appropriate setting for obesity treatment (especially if no concomitant disease is present), that time is lacking for patient visits, that reimbursement systems are inappropriate, that distinct and evidence-based guidelines need to be improved, and that patient motivation to change is low [7,8,12]. Male staff emphasised to a higher degree than female staff that there is a lack of guidelines and evidence. This may reflect that men to a higher degree explain lack of success in obesity treatment in terms of external (organisation) rather than internal (personal competence) causes. The conception that primary health care is not necessarily the best arena for the prevention and treatment of obesity was more evident among GPs than among DNs. This is in line with the finding of Mercer and Tessier [12] to the effect that GPs were more negative as to their role in obesity treatment than were nurses.

Staff in this study (mainly female GPs) emphasised the need for respectful treatment and individual solutions, and showed an understanding of the difficulty of changing lifestyle. This replicates what has been found in previous qualitative studies. Brown and Thompson [14] and Epstein and Ogden [8] reported that staff perceived the patient-provider relationship to be central to the improvement of obesity treatment. Patients who are informed and involved in decision-making have been found to be more adherent [20] and staff engaged in patient-centred care and make decisions together with their patients are in a better position to offer more individualised behavioural recommendations to their patients, resulting in better adherence [21]. Patients themselves have also asked for a more personalised approach to weight management, and for specific advice rather than broad statements on how to lose weight [22].

However, we also found that some staff experienced that, to motivate patients, they had to threaten them with a possibly fatal outcome, or at least inform them about the negative consequences of obesity. There is evidence in the literature that some patients view even mild warnings as scare tactics, with a negative impact on adherence [23]; others, however, regard warnings as encouraging and motivating, even essential to change [22]. It is important that GPs and DNs assess patient motivation and discuss what facilitates motivation individually. Previous studies have shown that patients report greater motivation and are more optimistic about weight loss than their GPs, but those who see obese patients more often are better at predicting patient motivation [24].

The findings of the present study regarding the staff's perception that some patients exhibit evasive behaviour, are untrustworthy when it comes to revealing their lifestyles and off-load their problems on to staff, are in line with previous research [8,12]. These conceptions were more strongly expressed by DNs than GPs in our study. One reason for this difference could be that the sort of behaviour in question may not appear until after a couple of sessions, whereby DNs are more likely to encounter it in that they often spend more time with patients than do GPs. The problem with these attitudes on the part of staff is that they may manifest themselves in encounters with patients, and that patients might thereby sense that staff do not trust them [25]. Previous studies have shown that patients' higher body mass index is associated with less respect from their doctors [26] and higher reporting of perceived discrimination in a health care setting [27]. Respect develops over time, therefore it would seem necessary that goals in obesity treatment should include continuity of care and long-term support, as indeed was emphasised by staff in our study.

The use of scare tactics and perceptions of patients as non-adherent might be due to staff's not being able fully to use or appreciate the strategies of motivational interviewing that are central to increasing patients' intrinsic willingness to change. Female GPs and DNs, and one male GP, were the only ones that considered that lack of self-confidence in changing lifestyle could be the reason for patients' not succeeding in losing weight. Patients' belief in their ability to make the necessary changes has been found to be important for behaviour change [28], and an understanding of this on the part of the health care provider seems crucial. This finding may suggest that male GPs and DNs are too quick to jump to a conclusion about what underlies some of their patients' non-adherence.

Staff stressed the need for more knowledge and skills in counselling, such as motivational interviewing, and also in basic nutrition and appropriate dietary interventions. The question is whether staff are being trained in the most updated methods of obesity treatment. Staff might, for example, also need skills directed towards helping patients to cope with their situation, because of the limited chance of losing weight going together with the stigma related to obesity. Coping strategies to deal with stigma have important implications for emotional functioning, and health care staff could assist in finding methods to help improve patients' daily functioning.

There seems to be a need for evidence-based guidelines which are easy to use and regarded as effective by staff. The use of guidelines is intended to improve the quality of treatment, and it has been found that nurses with no specific preparation or guidelines regarding obesity treatment were the ones who experienced most awkward in meeting obese patients [14]. However, a study of GPs found that awareness of the guidelines was associated with a more negative attitude towards obesity [6]. These contradictory findings suggest that, even when guidelines are available, they might not be user-friendly, updated and/or integrated appropriately into primary care.

Staff in the present study emphasised the importance of having an active interest in obesity treatment. To further enhance work at primary care centres, it might be necessary for every unit to appoint specific staff to take responsibility for structuring activities and engaging others regarding obesity treatment, organised perhaps in the same way as for diabetic patients. Findings from the Counterweight Programme [29] show, for example, that staff perceived that involvement and a sense of ownership, alongside a clear understanding of programme goals, are important factors in the effective implementation of weight management in primary health care.

### Limitations and Strengths

There are certain limitations to consider when it comes to interpreting the findings of the present study. Firstly, there were quite a number of health care centres which refused participation, for which reason it is possible that the participants came from centres which had taken an active interest in weight management. The health care centres included, however, were situated in both affluent and poor areas of Stockholm, and were both large and small. The demographic characteristics of non-participants and participants are unfortunately not known. Secondly, the number of male participants was low (one third). However, owing to the limited number of males eligible from the nurse population and to the fact that previous research has not been able to recruit male nurses, this study makes an important contribution to the field. Thirdly, some of the participants were recommended by their medical head, and it is likely that those with a more positive attitude were chosen. However, the findings display very rich descriptions of conceptions; both negative and positive views were expressed, and participants were not afraid of raising difficult topics.

All participants were asked the same questions, and all interviews and analysis were performed by the first author (LMH), who is a nutritionist. However, the possibility cannot be excluded that the background of the interviewer prompted participants to focus more on issues related to healthy diet and physical activity. To attain greater rigour, the conceptions and descriptive categories derived were scrutinised at each step by the third author (GA), who is experienced in phenomenographic analysis. The findings were constantly discussed in depth during the analysis by two of us (LMH, GA) until agreement was reached.

### Implications for Clinical Practice and Future Research

It is likely that low confidence of staff in treating obesity means that obesity in primary care has low priority, and their belief that patients are not motivated produces a moral dilemma for GPs and DNs. How should they prioritise their work? Is obesity the responsibility of the patient or of the health care system? Obesity treatment in primary health care, though, has the potential of being much more effective than it currently is, and the GPs and DNs in the present study touched upon many organisational aspects that need improvement. For example, obesity must be recognised as an important issue at all levels of the health care system. It also seems warranted to promote competence in motivational interviewing and evidence-based treatments, and also to increase awareness of staff's negative views on patient attitudes. Additional ways to enhance care might be to adopt a team-based approach within each unit, with resources to enable continuity in care, and also to promote co-operation with other stakeholders, such as social welfare authorities, commercial weight-loss organisations and specialist obesity units.

Finally, the gender- and profession-based differences which were found are somewhat difficult to interpret and therefore deserve further investigation in larger quantitative studies. For instance, to our knowledge no one has compared both profession and gender aspects in the same study. Moreover, research should investigate the association between staff's negative perceptions of patients with obesity and their actual practices, which, in the long run, might have additional harmful effects on obese patients' health.

## Competing interests

The authors declare that they have no competing interests.

## Authors' contributions

LMH, FR and GA all participated in the design of the study. LMH conducted all the interviews, made the initial analysis of the interview transcripts and drafted the manuscript. LMH and GA had discussions about the analysis and reporting. FR and GA offered comments on the draft of the manuscript. All authors read and approved the final manuscript.

## Pre-publication history

The pre-publication history for this paper can be accessed here:

http://www.biomedcentral.com/1471-2296/12/7/prepub
